# Practice variations in indication, timing and outcome of Multiple Myeloma patients undergoing surgery for vertebral lesions – results from the European M2Spine study group

**DOI:** 10.1007/s11060-025-05085-y

**Published:** 2025-06-13

**Authors:** Vanessa Hubertus, Lennart Viezens, Martin Stangenberg, Anton M. Früh, Hanno S. Meyer, Raimunde Liang, Andreas Kramer, Christoph Orban, Johannes Kerschbaumer, Beate Kunze, Stefano Telera, Hannah Miller, Christian J. Entenmann, Emily J. von Bronewski, Charlotte Buhre, Leon-Gordian Leonhardt, Wolfgang Willenbacher, Irma Kvitsaridze, Dominik Laue, Matthias Pumberger, Theresa Keller, Güliz Acker, Jan Krönke, Igor-Wolfgang Blau, Ulrich Keller, Florian Ringel, Claudius Thomé, Bernhard Meyer, Peter Vajkoczy, Marc Dreimann, Julia Sophie Onken

**Affiliations:** 1 Department of Neurosurgery, Charité – Universitätsmedizin Berlin, Corporate Member of Freie Universität Berlin, Humboldt-Universität zu Berlin, Berlin Institute of Health, Charitéplatz 1, 10117 Berlin, Germany; 2 BIH – Berlin Institute of Health Charité Clinician Scientist Program, Berlin Institute of Health, Berlin, Germany; 3 Division of Spine Surgery, Department of Trauma and Orthopedic Surgery, University Medical Center Hamburg- Eppendorf, Hamburg, Germany; 4 Department of Neurosurgery, University Medical Center Hamburg-Eppendorf, Hamburg, Germany; 5 Department of Neurosurgery, Klinikum Rechts der Isar, Technical University of Munich, Munich, Germany; 6 Department of Neurosurgery, University Hospital Mainz, Johannes-Guttenberg-University Mainz, Mainz, Germany; 7 Department of Neurosurgery, Medical University of Innsbruck, Innsbruck, Austria; 8 Spine Center Markgröningen, Markgröningen, Germany; 9 IRCCS Regina Elena National Cancer Institute, Rome, Italy; 10 Department of Internal Medicine, Hematology and Oncology, Comprehensive Cancer Center Innsbruck (CCCI), Austrian Comprehensive Cancer Network (ACCN), Tyrolean Cancer Research Center (TKFI), Medical University of Innsbruck, & Syndena GmbH, Connect to Cure, Innsbruck, Austria; 11 Department of Radiation Oncology, Medical University of Innsbruck, Innsbruck, Austria; 12 Department of Orthopedic and Trauma Surgery, Campus Benjamin Franklin, Charité - Universitätsmedizin Berlin, Corporate Member of Freie Universität Berlin, Humboldt-Universität zu Berlin, Berlin Institute of Health, Berlin, Germany; 13 Department of Orthopedic and Trauma Surgery, Center for Musculosceletal Surgery, Campus Berlin Mitte and Campus Virchow, Charité – Universitätsmedizin Berlin, Corporate Member of Freie Universität Berlin, Humboldt- Universität zu Berlin, and Berlin Institute of Health, Berlin, Germany; 14 Institute of Biometry and Clinical Epidemiology, Charité – Universitätsmedizin Berlin, Corporate Member of Freie Universität Berlin, Humboldt-Universität zu Berlin, and Berlin Institute of Health, Berlin, Germany; 15 Department of Radiation Oncology and Radiotherapy, Charité – Universitätsmedizin Berlin, Corporate Member of Freie Universität Berlin, Humboldt-Universität zu Berlin, Berlin Institute of Health, Berlin, Germany; 16 Department of Hematology, Oncology, and Cancer Immunology, Charité – Universitätsmedizin Berlin, Corporate Member of Freie Universität Berlin, Humboldt-Universität zu Berlin, Berlin Institute of Health, Berlin, Germany; 17 German Consortium for Translational Cancer Research, DKTK, Part of the German Cancer Research Centre, Berlin, Germany

**Keywords:** Multiple myeloma, Vertebral column lesions, Chronic vertebral pain, Surgical decision-making, Surgical complications

## Abstract

**Purpose:**

Painful vertebral lesions are pathognomonic in Multiple Myeloma (MM). While non-surgical management is generally preferred, some patients ultimately require surgical intervention. Here we describe the largest European cohort of MM patients with vertebral lesions to examine the practice variations of spine surgery in means of indication, timing and outcome.

**Methods:**

This study included patients with MM vertebral lesions enrolled in the European M2Spine Registry (DRKS00033326) at seven European academic spine centers between 2005 and 2023. Retrospective analysis evaluated epidemiological, clinical, and oncological treatment, focused on surgical management. Uni- and multivariate analyses identified factors associated with a decision towards spine surgery, including transitions from initially intended non-surgical approaches.

**Results:**

704 patients were enrolled and 493 (70%) surgically treated. Main indications for surgery were refractory vertebral pain (41%) and neurological deficits (22%). Radiological and clinical parameters indicating spinal instability as assessed retrospectively were present in 32% but associated with surgical management in only 43%. 338 patients (48%) underwent surgery during early disease stage, while 110 (16%) received delayed surgery (median: 42 months, range: 12–306 months). Statistical analysis revealed lower MM grading (ISS) at diagnosis (*p* < 0.001), and a new onset of neurological deficits (*p* < 0.001) as the most significant indicators for a cross-over from intended non-surgical to surgical treatment. Of the 78% of patients available for neurological follow up, 94% of surgically treated patients showed an improved or stable neurological status after a median of 45 months.

**Conclusion:**

Surgical intervention proved to be a viable option for patients with refractory pain and neurological deficits. Data from future prospective studies are necessary to evaluate the clinical trajectory of surgical and non-surgical treatment, and to ultimately provide evidence-based surgical treatment guidelines for MM patients.

**Supplementary Information:**

The online version contains supplementary material available at 10.1007/s11060-025-05085-y.

## Highlights

- **Background:** Vertebral column lesions are a common manifestation of Multiple Myeloma (MM), often resulting in chronic vertebral pain and neurological deficits. While non-surgical management is universally preferred, certain clinical scenarios, such as refractory pain or progressive neurological deterioration, may necessitate surgical intervention. However, there is a lack of robust data on the safety and outcomes of surgical interventions in this patient population.
- **Study aim:** Description of the largest European cohort of patients with MM vertebral column lesions to examine the practice variations of spine surgery in means of indication, timing and outcome.
- **Key results:** Of the 704 enrolled patients, 70% received surgical treatment. While 338 (48%) patients underwent surgery during the early stages of disease, 110 (16%) received surgery either later during primary myeloma therapy or after completion of distinct treatment phases. Main reasons for surgery in all patients were refractory pain and a new onset of neurological deficits. Of all cases with radiological and clinical parameters indicating spinal instability, 43% received surgical management. Surgical technique was diverse, with a postoperative complication rate of 10%. There was no difference in complication rate and outcome based on timing of surgery. Of the patients available for neurological follow up, 94% showed an improved or stable neurological status (Mc Cormick scale) after a median of 45 months.
- **Meaning:** Spine surgery in MM patients is feasible and associated with moderately low complication rates. Notably, the timing of surgery - whether early, during chemotherapy, or post-chemotherapy - had no impact on surgical complication rates. Interestingly, radiological and clinical indicators of spinal instability prompted surgical management in only a limited proportion of cases, emphasizing the role of symptom-driven rather than instability-driven decision-making in this cohort.

**Supplementary Information:**

The online version contains supplementary material available at 10.1007/s11060-025-05085-y.

## Introduction

Multiple Myeloma (MM) is a plasma cell malignancy associated with significant clinical complications, including bone fractures, anemia, renal insufficiency, and hypercalcemia. 80% of MM patients develop bone-related complications over the course of their disease, 50% of which are caused by vertebral column lesions, with the consequence of vertebral fractures, chronic vertebral pain, and significant risks of neurological deficits [1–7]. The compromised structural integrity of the bone, combined with an increased risk of surgical site infections in these immunocompromised patients, contributes to a widespread reluctance to consider surgical intervention even in cases with severe symptoms [1, 6, 8–14]. Additionally, MM is considered sensitive to radio- and chemotherapy, allowing vertebral lesions with or without epidural tumor infiltration to be effectively [1, 9, 14–21]. Consequently, vertebral column lesions in MM patients are predominantly managed non-surgically.

However, there are MM patients where non-surgical treatment does not suffice. In cases where substantial vertebral pain remains, cement augmentation as a minimally invasive approach has been proven to effectively reduce back pain and restore the patients` independence in daily living [1, 11, 22–25]. Cement augmentation however lacks the ability to restore spinal stability, prevent secondary deformities, or improve neurological deficits [26–31]. In such cases, surgical approaches like posterior instrumentation and fusion combined with decompression of neural structures represent the mainstay of surgical management from a spine surgeon`s perspective [26, 32–41]. In metastatic spine diseases (MSD), standardized scores like the Spinal Instability Neoplastic Score (SINS) and the Bilsky score are established to reliably guide surgical decision-making [35, 36, 39, 42–44]. Comparable data and established surgical treatment guidelines for the management of MM-related vertebral lesions are lacking. This retrospective cohort study seeks to address key aspects of the surgical management of MM patients suffering from symptomatic vertebral column lesions, including indications, timing, and outcome. Drawing from a registry of 704 patients compiled by the European M2Spine Study Group, these findings provide insights into the practice variations of current MM vertebral column lesion treatment, and the lack of evidence-based surgical treatment guidelines for MM patients.

## Methods

### Study cohort and clinical data

Electronic patient records were collected at seven tertiary academic spine centers across Europe (Germany, Austria, and Italy), participating in the multicentric European M2Spine Registry (German Clinical Trial Registry, DRKS00033326). Records were retrospectively screened for patients with diagnosis codes “Multiple Myeloma” (ICD-10 C90) and “vertebral column lesion” (ICD-10 C79), who were treated between 2005 and 2023 at the participating study centers. Database lock was in December 2023. Patients were treated by an interdisciplinary expert team of spine surgery, hematology and oncology and radio-oncology. After anonymization, data was transferred to the primary study center for analysis. Clinical data collection was conducted during standard treatment and in a retrospective fashion.

### Inclusion criteria

All MM patients with symptomatic vertebral column lesions, patient age ≥ 18 years, and treatment (non-surgical or surgical) at one of the participating study centers between 2005 and 2023.

### Exclusion criteria

Patients aged < 18 years, vertebral column lesions due to other neoplastic entities, solitary plasmacytoma, or intraspinal, intradural MM or other neoplastic lesions without vertebral body affection.

### Outcome parameters

The primary outcome parameters were complication rate of spine surgery, and neurological outcome. Secondary outcome parameters included conservative treatment failure and factors contributing to delayed surgical intervention.

### Data collection

Detailed demographic, medical, hematological, surgical and radiological data which were available through routine documentation during the patient`s treatment were collected retrospectively, recorded in a standardized, and stored in an anonymized fashion. The following scores were recorded: ECOG (Eastern Cooperative Oncology Group), Karnofsky (KPS) and ASA (American Society of Anesthesiology), as assessed at initial presentation and as available during follow-up. Additional oncological data comprised of MM type and subtype, CRAB criteria (hyper*c*alcemia, *r*enal failure, *a*nemia, or lytic *b*one lesions), and stage of disease (ISS – International Staging System) at initial MM diagnosis. Additionally, records and available imaging data (CT, MRI) were assessed, precisely characterizing each vertebral column lesion. Spinal stability was retrospectively evaluated as available on CT/MR imaging based on radiological and clinical criteria in accordance with the Spinal Instability Neoplastic Score (SINS) [39]. The SINS is a numerical score (1–18 points) summarizing information on the vertebral lesions` location (junctional, mobile, semi-rigid, rigid spine), associated vertebral pain (pain with loading of the spine, non-mechanical pain, painless lesion), the lesions` structure (lytic, blastic, mixed), the grade of pathologic vertebral body collapse (> 50%, < 50%, no collapse with > 50% of the vertebral body involved, none of the above), and posterolateral involvement (bilateral, unilateral, none of the above). Based on the overall sum score, lesions were categorized as stable (SINS 1–6), potentially unstable (SINS 7–12) and unstable (SINS 13–18) [39, 42]. Information on the timepoint of diagnosis of vertebral lesions, associated symptoms and the acuteness of clinical presentation were recorded. The modified McCormick Scale (1–5) was assessed to grade the patient`s ambulatory status and need for assistance. Each lesion`s clinical course and management was monitored during follow-up, as available. Surgical information included indications for surgery, time from lesion detection to surgical intervention, and type of surgery. Surgical strategies were chosen by the spine surgeons in charge at the participating centers.

### Data management and statistical analysis

Study data were collected and managed using a multicentric REDCap^®^ (Research Electronic Data Capture) database. Statistical analysis and data visualization was performed using GraphPad Prism 9 (GraphPad Software, La Jolla, CA, USA), SAS Version 9.4, R with R studio Version 2023.06 (packages: ggplot2, dplyr, redxl), and biorender.com. For the descriptive analysis of differences between two groups in nominal variables, unpaired T-test was used for normally distributed data, and Mann-Whitney test for non-normally distributed data. For the descriptive analysis of more than two groups, a one-way ANOVA test combined with Bonferroni’s multiple comparison test was performed for normally distributed data, and a Kruskal Wallis test with Dunn`s test for multiple comparisons for non-normally distributed data. Categorial variables were tested using Xi-Square testing. For survival analysis, Kaplan-Meier survival curves were obtained, with testing for significant differences using the log-rank test. All statistical analyses were exploratory in nature, with p-values interpreted descriptively.

### Ethical statement

This study was conducted in accordance with the ethical principles of medical research involving human subjects according to the Declaration of Helsinki and its later amendments. Clinical data were assessed and anonymized for patients’ confidentiality. Ethical approval (EA4/063/20) was granted by the institutional ethics board of the local ethics committees of the primary study center and all participating study centers. Informed patient consent was not required according to the Ethical approval. Clinical data collection was conducted during standard treatment and in a retrospective fashion.

## Results

### Description of the entire study population

**Fig. 1:**
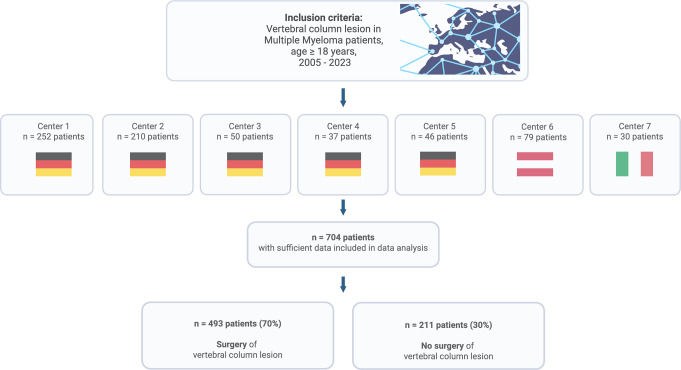
Overview of study inclusion criteria and study recruitment. Figure created with Biorender.com

A total of 704 patients met the inclusion criteria and were enrolled in the European M2Spine Registry. Included patients were treated at seven participating European institutions. Mean patient age at inclusion was 66 years (range: 32–89), with male predominance (62% male, 38% female). Patients presented in a wide range of overall clinical status as measured by the KPS (mean 70, range 30–100). (*Overview of study recruitment*: Fig. 1. *Detailed clinical data*: Table 1).

The thoracic spine was the most frequently affected region (*n* = 490, 30% of all lesions), followed by the lumbar (*n* = 382, 23%) and cervical spine (*n* = 227, 14%). A disseminated pattern involving two or more spine regions was frequent (*n* = 430, 50%). Most lesions showed radiological signs of potential instability (*n* = 320, 48%) or obvious instability (*n* = 215, 32%) according to SINS, when applied retrospectively.

At the time of initial MM diagnosis, 346 patients (50%) were concurrently diagnosed with vertebral column lesions. In the mean six months (range: 0-348) passed between initial MM diagnosis and the first detection of vertebral column lesions. Regarding clinical presentation, most patients (*n* = 527, 87%) suffered from vertebral pain at the lesion`s location. Neurological deficits of any kind were apparent in 119 patients (18%), with motor deficits in 89 (12%). Most patients were ambulatory with or without minor assistance (mod. McCormick 1: *n* = 340, 71%, 2: 103, 21%), while severe impairment and need for assistance was the case only in a minority (mod. McCormick 3–5: *n* = 39, 8%). Few patients presented with acute-onset clinical problems like acute pain or acute neurological deficits (*n* = 69, 11%, detailed data overview: Table 1).

**Table 1:** Characteristics of the study population

Item	Study cohort
Total number of patients included	704
Mean patient age (range)	66 (32–89)
Sex, n (%)	Male 436 (62), female 270 (38)
ASA score, n (%)	1–23 (5), 2–194 (40), 3–242 (50), 4–21 (4)
KPS, mean (range)	71 (30–100)
ECOG, n (%)	0–109 (22) 1–226 (46) 2–104 (21) 3/4–48 (11)
MM type and subtype, n (%)	IgG 346 (53) Light Chain 182 (28) IgA 106 (16) IgM, IgE or IgD 13 (2) Subtype: Kappa 405 (64), Lambda 184 (29), not specified 43 (7)
ISS at initial MM diagnosis, n (%)	1–136 (32) 2–165 (40) 3–117 (28)
CRAB positive at initial MM diagnosis, n (%)	CRAB positive 562 (86), CRAB negative 92 (14)
Bisphosphonate treatment, n (%)	407 (74)
First-tier oncologic therapy regiment applied, n (%)	High-dose therapy with maintenance 281 (48) High-dose therapy without maintenance 163 (28) Non-high-dose therapy 105 (18) Therapy-naïve 33 (6)
Stem-cell transplantation, n (%)	246 (42)
Time from initial MM diagnosis to MM vertebral lesion in months, mean (range)	6 (0-348)
Diagnosis due to MM vertebral lesion, n (%)	346 (50)
Localization of MM vertebral lesion (spine region affected), n (%)	Cervical 227 (14), Thoracic 490 (30), Lumbar 382 (23), Sacral 115 (7)
Stability of MM vertebral lesions*, n (%)	Stable 131 (20) Potentially unstable 320 (48) Unstable 215 (32)
Vertebral pain due to MM vertebral lesion, n (%)	527 (87)
Neurological deficits due to MM vertebral lesion, n (%)	119 (18) Vegetative 30 (%), Sensory 97 (%), Motor 89 (%)
Modified McCormick score, n (%)	1–340 (71) 2–103 (21) 3–25 (5) 4/5–14 (3)
Acuteness of complaints regarding MM vertebral lesion, n (%)	Acute 69 (11), Subacute 111 (18) Non-acute 404 (65), No complaints 35 (6)
Radiotherapy for MM vertebral lesion, n (%)	328 (47)
Progressive disease during available follow-up, n (%)	183 (34)
Available follow-up in months, median (range)	60 (1-348)
Overall survival in months, median (range)	36 (1-348)

ASA = American Score of Anesthesiology, KPS = Karnofsky Performance Scale, ECOG = Eastern Cooperative Oncology Group score, CRAB criteria = Calcium, Renal insufficiency, Anemia, and Bone lesions, MM = Multiple Myeloma, Ig = Immunoglobulin, ISS = International Staging System for Multiple Myeloma, * spinal stability assessed in accordance with Spinal Instabilty Neoplastic Score (SINS) criteria

### Surgical cohort

**Fig. 2:**
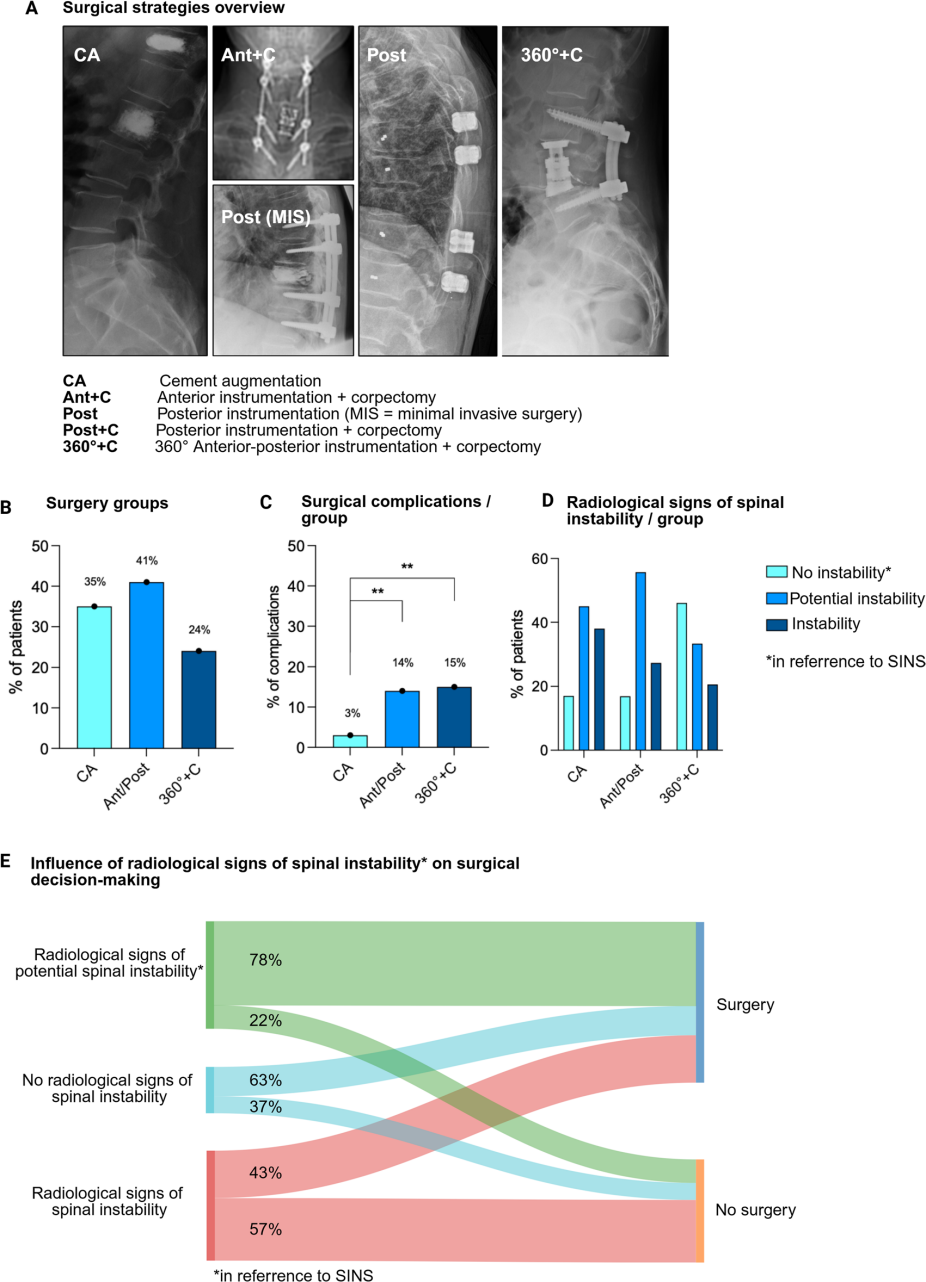
**A** Overview of surgical strategies. Abbreviations: CA = Cement augmentation (vertebro-/kyphoplasty), Ant + C = Anterior instrumentation with corpectomy, Post = Posterior instrumentation (MIS = minimal invasive surgery), Post + C = Posterior instrumentation with corpectomy, 360°+C = 360° Anterior-posterior instrumentation with corpectomy. **B** Overview of the Percentage of patients treated within the surgical groups (CA, Ant/Post including Ant + C, Post and Post + C, 360°+C), and **C** of the associated surgical complication rates, with ** = *p* < 0.01. **D** Surgical strategies stratified by degree of spinal stability. **E** Influence of spinal stability on applied treatment. * Spinal stability assessed in accordance with Spinal Instability Neoplastic Score (SINS) criteria

Main indications for surgery in the whole cohort were refractory pain (41%) and neurological deficits (22%). Other less frequent reasons for surgical interventions were the necessity of histology, or the treating physician`s fear of spinal instability. Surgical strategies varied widely, reaching from minimally invasive procedures like cement augmentation (35%), to anterior/posterior instrumentation and fusion techniques (41%) with or without 360-degree vertebral body replacement (24%). Overall surgical complication rate was 10%, while depending on the complexity of the performed surgery (Cement augmentation = 3%, instrumentation = 14%, 360° instrumentation with corpectomy = 15%, *p* < 0.01, Fig. 2, A-C). The presence of radiological signs of spinal instability as assessed retrospectively was not consistently associated with surgical intervention (78% vs. 43%) or certain surgical strategies, reflecting a significant practice variation in the management of MM vertebral spine lesions *(*Fig. 2D + E).

**Fig. 3:**
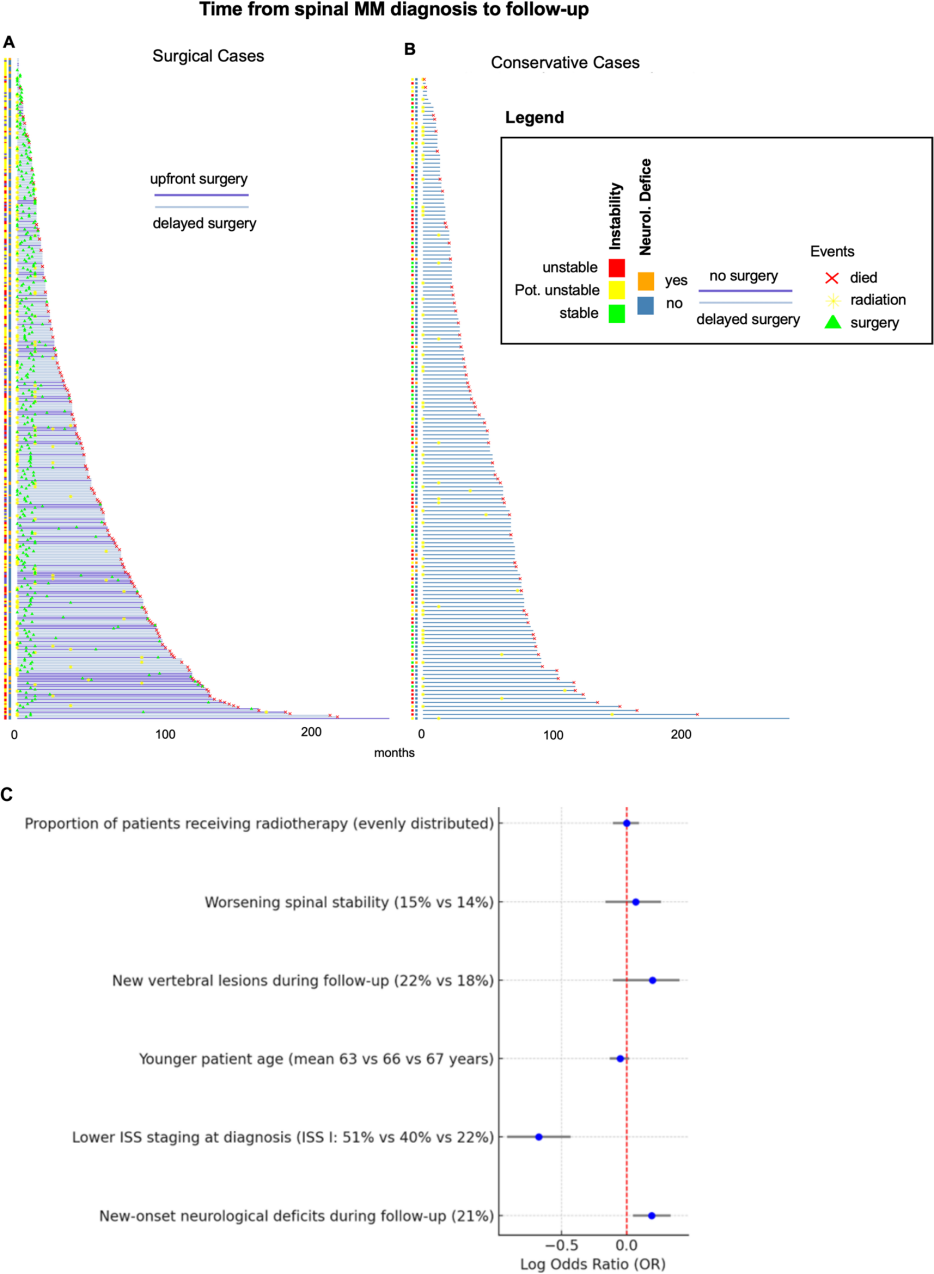
Swimmer Plot illustration the clinical course and timing of treatment starting from the time of fist diagnosis of MM vertebral lesion to the latest available follow-up or death of the patient, in **A** for all patients treated with surgery, and in **B** for all conservatively treated patients. **C** Forest plot: Odds ratio of factors associated with delayed surgery in MM patients

In respect to surgical timing, 338 patients (75%) were treated with upfront surgeryat the time of initial diagnosis of the vertebral column lesion. 110 patients (16% of the study population, 25% of all surgical patients) were opted for non-surgical management initially and received surgery at a later timepoint, with a mean delay of 42 months (range: 12–306). Detailed data on the clinical course in the surgical and non-surgical groups are presented for each individual in Fig. 3. Patients’ characteristics in the upfront surgery, delayed surgery, and non-surgical treatment groups are given in Table 2.

Factors statistically associated with delayed surgical treatment were the development of new-onset neurological deficits during clinical follow-up (21%), a lower ISS staging at diagnosis (ISS stage I 51% vs. 40% in the non-surgical group and 22% in the upfront surgery group, *p* < 0.001), and a tendency towards younger patient age (mean age 63 vs. 66 years in the non-surgical group and 67 years in the upfront surgery group, *p* = 0.021). In the comparison of the delayed surgical with the non-surgical group, no difference in the occurrence of new vertebral lesions during follow-up (22% vs. 18%, *p* = 0.63), or a worsening of spinal stability of previously known lesions (15% vs. 14%, *p* = 0.969) existed. The proportion of patients receiving radiotherapy was evenly distributed across all groups (Fig. 3C).

Complication rates were similar between the two groups, with 9% in the upfront surgery group and 10% in the delayed surgery group. Reoperation rate however was 1.67 times higher in the upfront surgery group (10%) compared to the delayed surgery group (6%). Main surgery-associated complications were surgical site infections, hematoma, and hardware failure. At latest follow up, 94% of patients where neurological follow-up was available (385 patients, 78%, median follow-up = 44.6 months) displayed a stable or improved neurological status after surgery, as assessed by the modified Mc Cormick scale. Patient`s median overall survival was longest in the delayed surgical group (median 60 months, range: 12–264), while comparable between the upfront surgical and the non-surgical groups (median 30 months, range: 1-348, vs. 36 months, range: 1-120, *p* = 0.054, Table 2).

**Table 2:** Univariate subgroup analysis comparing upfront surgical (i), delayed surgical (ii), and non-surgical (iii) management of multiple myeloma vertebral column lesions

Item	Upfront surgery (i)	Delayed surgery (ii)	No surgery (iii)	*p*-value
No. patients (% of whole)	338 (48)	110 (16)	211 (30)	N/A
Median time to surgery in months (range)	6 (0–11)	42 (12–306)	N/A	N/A
Mean patient age (range)	67 (37–89)	63 (25–87)	66 (32–88)	**0.021** (i vs. ii, ii vs. iii)
Sex, n (%)	Male 206 (60) Female 134 (40)	Male 73 (66) Female 38 (34)	Male 129 (61) Female 84 (39)	0.592
Mean KPS (range)	72 (30–100)	73 (40–100)	70 (30–80)	0.305
ISS, n (%)	1–45 (22) 2–101(49) 3–60 (29)	1–34 (51) 2–21 (31) 3–12 (18)	1–52 (40) 2–38 (29) 3–42 (32)	**< 0.0001**
Vertebral pain, n (%)	284 (90)	79 (88)	135 (82)	0.053
Neurological deficits, n (%)	76 (23)	21 (21)	14 (8)	**< 0.0001** (i vs. iii, ii vs. iii)
Spinal stability categories of MM vertebral lesions, (in reference to SINS), n (%)	Stable 59 (18) Pot. unstable 189 (57) Unstable 85 (26)	Stable 22 (20) Pot. unstable 43 (39) Unstable 46 (41)	Stable 47 (25) Pot. unstable 65 (35) Unstable 173 (39)	**< 0.0001**
Median SINS per patient (range)	9 (3–18)	11 (4–16)	10 (4–18)	0.078
Progressive spinal instability during follow-up, n (%)	36 (15)	13 (15)	20 (14)	0.969
New vertebral lesion during follow-up, n (%)	51 (21)	19 (22)	25 (18)	0.63
Radiotherapy of vertebral lesion, n (%)	160 (47) *Adjuvant 135 (40)*	55 (50) *Adjuvant 36 (33)*	93 (44)	0.561
Surgical complications, n (%, *details*, ≥ *1 complication per patient)*	33 (10), *SSI 15*,* Hematoma 6*,* HWF 7*,* Medical 5*	10 (9), *SSI 6*,* Hematoma 3*,* HWF 3*,* Medical 2*	N/A	0.856 (i vs. ii)
Reoperations, n (%)	20 (6)	11 (10)	N/A	0.192 (i vs. ii)
Mod. McCormick, median (range)	2.6 (1–5)	2.3 (1–5)	2.5 (1–5)	0.935
Follow-up in months, median (range)	43 (0-348)	59 (0-240)	46 (0-276)	**0.0009** (i vs. ii, ii vs. iii)
Overall survival in months, median (range)	30 (1-348)	60 (12–264)	36 (1-120)	0.054

No. = Number of, KPS = Karnofsky Performance Scale, SINS = Spinal Instability Neoplastic Score, MM = Multiple Myeloma, SSI = Surgical Site Infection, HWF = Hardware Failure, C = Cervical Spine, Th = Thoracic Spine, L = Lumbar Spine, S = Sacral Spine, Subgroups: i = upfront surgery, ii = delayed surgery (non-surgical treatment failure), iii = no surgery, % of whole with 100%=704 patients. Statistical analysis: Means per group were compared using one-way ANOVA with Bonferroni’s correction, or Kruskal-Wallis-Test with Dunn`s test for multiple comparisons, as appropriate depending on Shapiro-Wilk-test for Gaussian distribution. Categorial variables were tested with Xi^2^ and Fisher`s exact test. Statistical significance was tested in an exploratory fashion, with *p* < 0.05 set as statistically significant

## Discussion

The principal novel findings of this study are that spine surgery in selected Multiple Myeloma patients can be performed safely with a low complication rate (10%) and mostly stable or improved neurological function at a median follow-up of 45 months (clinical follow-up rate 78%). Notably, the timing of surgery - whether early, during MM therapy, or after the completion of distinct treatment phases - had no impact on complication rates. Interestingly, radiological indicators of spinal instability as assessed retrospectively prompted surgical management in only a limited proportion of cases, emphasizing the role of symptom-driven rather than instability-driven decision-making in this cohort.

Of all included patients, 493 (70%) underwent surgical intervention. Despite the presence of refractory vertebral pain, non-surgical management was initially favored for 211 (30%) patients. However, a significant subset of patients (*n* = 110, 16% of the study population, 25% of all surgical cases) transitioned to surgery at a later point, after a median of 42 months following the initial vertebral lesions` detection. Surgical indications for these patients often included a new onset of neurological deficits due to disease progression. In the whole study population, refractory pain and the occurrence of neurological deficits were the driving factors to perform surgery.

The most common surgeries performed in this series included decompressive procedures and fusion techniques aimed at both relieving neurological symptoms and addressing mechanical instability. The overall complication rate was low with 10%, which is a favorable outcome considering the frail condition of many MM patients and compares well with published data for MSD [26, 40, 45–54]. The timing of surgery did not influence the occurrence of surgical complications, as similar rates were observed in both the upfront (10%) and delayed surgery groups (9%). As was to be expected, surgical complication rates depended on the complexity of the performed surgery. Cement augmentation was associated with a very low complication rate (3%), while decompression and instrumentation prompted a complication rate of 15%, and 360° instrumentation with corpectomy was associated with a rate of 16% (*p* < 0.01). In comparison, a large national registry of patients with MM or plasmacytoma vertebral lesions treated with surgery in the U.S. (*n* = 14,687) recently reported surgical complication rates of 22% (Overall), 27% (Decompression), 23% (Stabilization), 22% (Cement Augmentation) [55]. 

At the time of initial MM diagnosis, 346 patients (50%) in this study were concurrently affected from vertebral column lesions, while a subset of those patients suffered from symptomatic vertebral lesions even before MM diagnosis was proven. This fact serves as one explanation for the rather high rate (24%) of patients treated with 360° instrumentation and corpectomy in this study. Internationally, it is preferred to treat MM patients with less invasive surgical strategies than primary bone tumors or MSD, which highlights cement augmentation and decompression techniques, or minimally-invasive instrumentations as preferable in this multimorbid patient collective with an increased risk of surgical site infections due to a compromised immune system [1, 6, 8–14]. 

Interestingly, reoperation rate was 1.67-fold higher in the upfront surgery group (10%) compared to the delayed surgery group (6%). This finding underscores the potential advantage of carefully evaluating surgical timing in MM patients. Factors associated with delayed surgical management were younger aged patients, a lower ISS score, and a new onset of neurological deficits, but notably not spinal instability. According to these results, younger patients or patients with a lower tumor scoring at diagnosis might tolerate initial non-surgical management better but may ultimately progress to a point where surgical intervention becomes necessary. The onset of neurological deficits in this study foremostly influenced the decision to transition from non-surgical towards surgical management. This underscores the importance of close clinical monitoring and standardized imaging protocols for patients with non-surgically treated MM vertebral lesions, particularly those at a higher risk for disease progression [1, 4, 56–58]. However, delayed surgical intervention might allow for better systemic disease control, bone consolidation, and optimization of the patient’s overall condition, reducing the likelihood of additional surgical procedures.

Radiological signs of spinal instability as assessed retrospectively were not consistently associated with the indication for surgical intervention in this study, and cases of delayed surgery showed no progression of preexisting spinal instability. This suggests that bone consolidation achieved through systemic and/or radiotherapy in MM patients seems to be robust, shifting the focus of surgical indications towards more symptom-driven criteria as compared to MSD. Standardizing treatment algorithms and defining clear criteria for surgical intervention in MM patients will in the future help to reduce treatment variability and enhance consistency in patient care and outcomes [6, 9, 10, 17, 58–60]. 

### Limitations

This retrospective multicenter cohort study has several limitations. First, a selection bias for surgically treated patients may exist, as the recruitment was predominantly completed by the surgical disciplines and referral of patients for surgery by non-surgical disciplines might be centered on “worst cases”. Second, despite the interdisciplinary care provided, involving hemato-oncologists, radio-oncologists, and spine surgeons, a certain proportion of patients received hematological therapies and/or radiotherapy at institutions outside the study centers. Consequently, clinical details regarding these treatments, such as radiotherapy plans, doses, and techniques, may be incomplete or unavailable, limiting the comprehensiveness of the collected data. Third, the study’s design, encompassing seven academic centers in Germany, Austria, and Italy, and the fact that surgical indications and strategies were determined independently by treating spine surgeons at each center, introduces some treatment heterogeneity [1, 9, 14–21, 54]. 

## Conclusion and outlook

While this study provides valuable insights into surgical indications, timing, and outcomes, critical questions remain about the impact of treatment modality and timing on health-related quality of life (HRQoL), pain relief, prevention of neurological deterioration, and the overall clinical course.

While the overall therapeutic strategies for patients with severe MM bone disease are widely discussed in multi-disciplinary tumorboards including all relevant specialties, future research should focus on prospective observational trials that comprehensively assess HR-QOL, pain scores, activity levels, pain medication usage, complication rates, and imaging findings. These studies should also aim to identify key factors that reliably predict which patients may benefit most from early versus delayed surgical intervention, with the ultimate goal to establish standardized, evidence-based treatment algorithms tailored to MM-related vertebral lesions, improving patient care and clinical outcomes while reducing variability in practice.

## Electronic supplementary material

Below is the link to the electronic supplementary material.

Supplementary Material 1

## Data Availability

Data is provided within the manuscript or supplementary information files.

## Abbreviations

ANOVA: Analysis Of Variance
ASA: American Society of Anesthesiology
CRAB: Hypercalcemia, Renal failure, Anemia, or Lytic Bone Lesions
CT: Computed Tomography
ECOG: Eastern Cooperative Oncology Group
HR-QOL: Health-Related Quality Of Life
HWF: Hardware Failure
ICD-10: International Statistical Classification of Diseases and Related Health Problems
Ig: Immunoglobulin
ISS: International Staging System
KPS: Karnofsky Performance Score
M2Spine Study Group: Multiple Myeloma Spine Study Group
MM: Multiple Myeloma
MRI: Magnetic Resonance Imaging
MSD: Metastatic Spine Disease
OS: Overall Survival
PFS: Progression-Free Survival
REDCap®: Research Electronic Data Capture
rISS: revised International Staging System
SINS: Spinal Instability Neoplastic Score
SSI: Surgical Site Infections

## Declarations

### Previous presentations

Parts of this work were presented at the Annual Congress of the German Spine Society (DWG) 2024, Nov 27-29, in Hamburg, Germany.
