# Supplementary material for: Practice variations in indication, timing and outcome of Multiple Myeloma patients undergoing surgery for vertebral lesions – results from the European M2Spine study group
Source: J Neurooncol. 2025 Jun 13;174(3):765–77. doi: 10.1007/s11060-025-05085-y (PMC12263761; doi:10.1007/s11060-025-05085-y)
Supplement: Supplementary file 1 — Supplementary Material 1 [file 11060_2025_5085_MOESM1_ESM.docx]

*
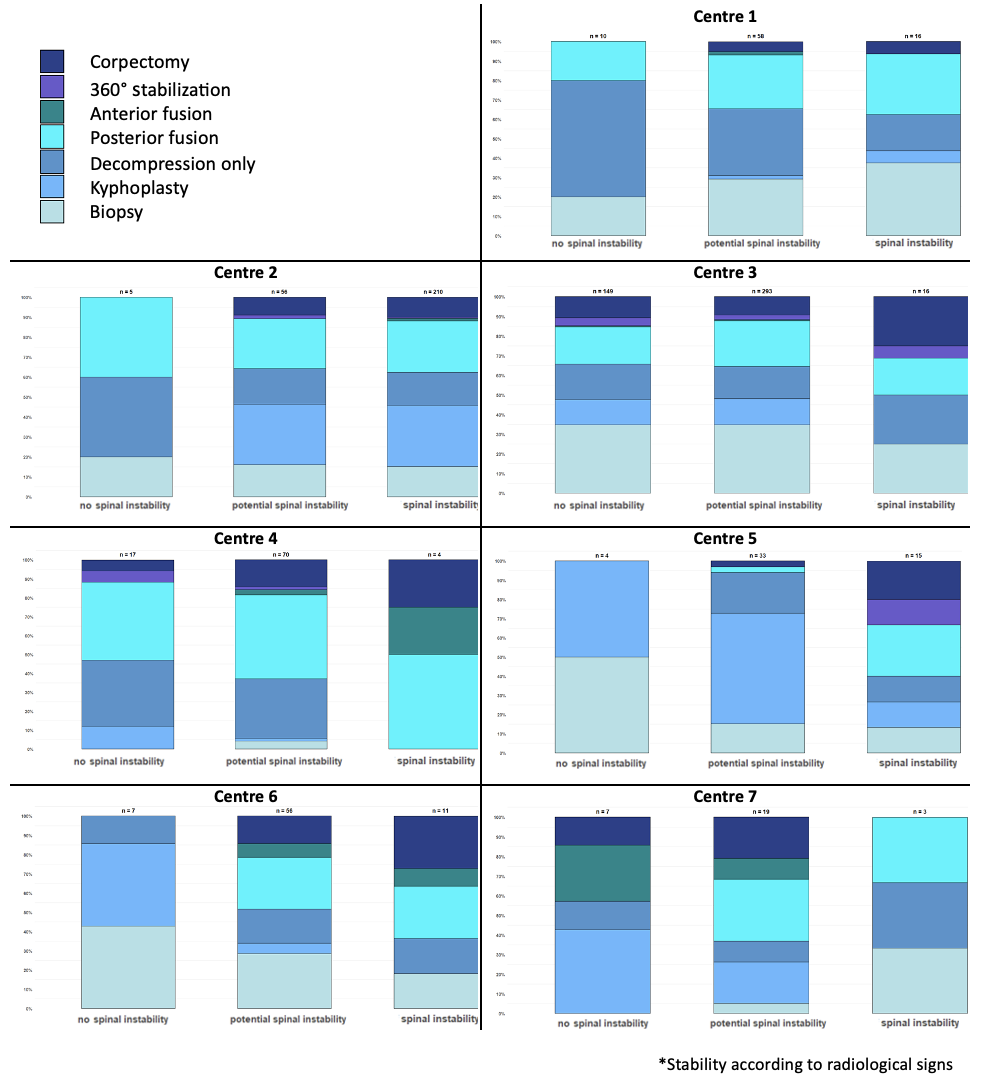
*

***Supplemental Figure 1:*** *This supplemental file highlights the large practice variations in surgical management in relation to the retrospectively assessed radiological and clinical parameters of spinal instability (in reference to the SINS) within each of the seven participating centers.*
